# Prevalence and associated factors of *Chlamydia trachomatis* and *Neisseria gonorrhoeae* among female commercial sex workers in Hawassa City, Southern Ethiopia

**DOI:** 10.1186/s12879-019-3698-8

**Published:** 2019-01-17

**Authors:** Alelign Tadele, Siraj Hussen, Techalew Shimelis

**Affiliations:** 1 Department of Medical Laboratory Science, Hawassa College of Health Sciences, South Nations and Nationalities Peoples Region, Hawassa, Ethiopia; 2 School of Medical Laboratory Science, College of Medicine and Health Sciences, Hawassa University, Hawassa, Ethiopia

**Keywords:** *Chlamydia trachomatis*, *Neisseria gonorrhoeae*, Female commercial sex workers, Hawassa, Ethiopia

## Abstract

**Background:**

*Chlamydia trachomatis* and *Neisseria gonorrhoeae* are the most common pathogens causing genital tract infections. Female commercial sex workers (FCSWs) are the key population to be affected by sexually transmitted infections (STIs). In Ethiopia, little is known about *C. trachomatis* and *N. gonorrhoeae* infections in most at risk population. Therefore, this study aimed to assess the prevalence of these bacterial STIs among FCSWs.

**Methods:**

A cross-sectional study was conducted at the confidential clinic in Hawassa City, Southern Ethiopia from January to April, 2017. A total of 338 FCSWs were selected using systematic random sampling technique and enrolled in the study. Information about socio-demography and associated factors was collected using structured questionnaires. Endocervical swab samples were also collected from the study participants and tested for *C. trachomatis* using rapid immunochromatography assay. Samples were also cultured to isolate *N. gonorrhoeae* according to the standard bacteriological method.

**Results:**

The prevalence of *N. gonorrhoeae* and *C. trachomatis* among FCSWs was 3.3% [95% confidence interval (CI): 1.5–5.3] and 6.8% (95% CI: 3.9–9.5), respectively. FCSWs who consistently practiced sex without condom in the last 6 months had 6.3 times (AOR 6.3; 95% CI 1.61–24.86, *P* = 0.008**)**, and 4.0 times (AOR 4.0; 95% CI 1.06–15.31, *p* = 0.040) higher odds of acquiring *N. gonorrhoeae* and *C. trachomatis* infections, respectively.

**Conclusion:**

The observed rates of *C. trachomatis* and *N. gonorrhoeae* infections among FCSWs warrant the need to strengthen intervention efforts. In this regard, screening FCSWs for the specified infections and improving the practice of condom use would be important.

## Background

*Neisseria gonorrhoea* and *Chlamydia trachomatis* are among the main pathogens causing sexually transmitted infections (STIs) [1, 2]. The World Health Organization (WHO) estimated that 50 million women worldwide were newly infected with *C. trachomatis*; of which, 34 million were in Sub-Saharan Africa and South/Southeast Asia [3]. With annual 82 million estimated cases globally, *N. gonorrhoeae* has been recognized as one of the major bacterial STIs. An estimated 17 and 27 million new cases annually occurred in Africa and in Southern Asia, respectively [1].

In women, urogenital *C. trachomatis* and *N. gonorrhoeae* infections cause broad spectrum of clinical manifestations, including urethritis, cervicitis, and pelvic inflammatory disease. If untreated, these infections may lead to serious complications, including ectopic pregnancy and tubal infertility [4]. However, over 50% of women with gonorrhea and 75% of women with *C. trachomatis* remain asymptomatic during the entire infection process. Thus, they are unaware of their infection to seek treatment, which facilitates further spread to their sexual partners [5, 6].

It was reported that chlamydial infection often occurred among women aged 15–29 years due tohost related factors such as the number of sexual partner, contraceptive use, sexual preference, and population mobility [5]. Female commercial sex workers (FCSWs) are at higher risk of contracting the infection [7], due to their social vulnerability and work related factors, like a history of having multiple sexual partners, inconsistent condom use, and a history of having STIs [8, 9]. FCSWs also link the spread of STIs to the general population through their clients [10].

In Ethiopia, the epidemiology of *C. trachomatis* and *N. gonorrhoeae* infection is not well described due to the asymptomatic nature of the infection and lack of laboratory diagnostic facilities. However, a hospital-based study that investigated women of reproductive age (15–49 years) reported prevalence of *C. trachomatis* and *N. gonorrhoeae* was 18.9 and 0.31%, respectively [11]. It would be important to study FCSWs to emphasize the need of strengthening intervention strategies that address the risk group. Therefore, this study aimed to assess the prevalence and associated risk factors of *C. trachomatis* and *N. gonorrhoeae* infections among FCSWs. The findings from this study help public health experts and other concerned bodies to design and undertake evidence-based interventions.

## Methods

### Study area and period

An institution-based cross-sectional study was conducted in Hawassa City, Southern Ethiopia from January to April, 2017. The city is situated on the shore of Lake Hawassa, in the Great Rift Valley, and located 275 km far south of Addis Ababa. Hawassa had a total population size of 343,175 people in the year 2014. FCSW is defined as any female, who practices sexual activity with a man in return for money, irrespective of the site of operation (i.e: street, bars, home and hotel). The only confidential clinic in the city is managed by the Family Guidance Association Ethiopia. The clinic has been serving FCSWs since August 2012, and provides comprehensive services including medical care, family planning, HIV voluntary counseling and testing (VCT), safe abortion, prevention of mother-to-child transmission (PMTCT), antenatal care (ANC), and management of STIs.

### Population

FCSWs, who received service in the confidential clinic during the study period, were the source population. FCSWs, who visited the confidential clinic during the study period, were eligible. FCSWs who attended the clinic more than once, those on STI treatment, and who were sick and unable to take part in the interview, were excluded.

### Sample size and sampling technique

The sample size was determined using a single population proportion formula to estimate the prevalence of *C. trachomatis* and *N. gonorrhoeae* infections among FCSWs. The sample size was calculated based on the following assumptions: prevalence of *N. gonorrhoeae* among FCSWs in Democratic Republic of Congo (7.8%) [12], 95% confidence interval, and 3% precision Further, considering a 10% non-response rate, the sample size was calculated to be 338. A systematic random sampling technique was used to recruit FCSWs attending the Confidential Clinic. Considering a four-month study period, a total of 592 FCSWs were expected to visit the clinic according to the clinic plan and the past 4 month’s performance document review. This estimate was divided by the sample size to determine the sample interval (k value), which would be 1.8. Thus, the 1st served client and every 2nd client afterwards was invited to participate in the study until the required sample size would be achieved.

### Data collection method

#### Data collection instrument and processing

##### Interview

A pre-tested and structured questionnaire was used to collect information on socio-demographic characteristics (age, residence, marital status, and educational level) and sexual related factors (consistent condom use in the last 6 months, age at first sex, years in sex work, self-reported history of STIs, number of client per week in the last 6 months, having regular partner) of study participants. Data was collected through face-to-face interview by two midwife nurses.

### Laboratory methods

#### Sample collection and transportation

The midwife nurses collected two endocervical swab samples from each study participant. One swab was collected for immediate testing of *C. trachomatis,* and the second swab was placed in Amies transport media and transported to Hawassa University Comprehensive Specialized Hospital using cold box for culturing of *N. gonorrhoeae.*

#### Isolation of *Neisseria gonorrhoeae*

Swab samples for *N. gonorrhoeae* were inoculated on modified Thayer-Martin agar and placed in a candle jar with 5–10% CO_2._ The inoculated plates were incubated at 37^0^c for 24–72 h. Bacterial growth was identified based on characteristic colony morphology, Gram reaction, and biochemical testing.

#### Testing of chlamydia antigen

Chlamydia antigen was detected by laboratory technologist in the confidential clinic using the onsite chlamydial rapid test kit (CTK Biotech, Inc., USA). This is a lateral flow chromatography immune assay based on double antibody-switched technique, which utilizes a unique part of the mouse monoclonal antibody to selectively identify *C. trachomatis* antigen in the specimen. Tests were performed according to the manufacturer instruction. A swab was inserted in to the sample extraction tube. Then four drops (200 μl) of buffer A were added and incubated at room temperature for 5 min. Further, four drops (200 μl) of buffer B were added and mixed by rotating the swab for 10 s and incubated for 1 min. Two drops of extract solution were dispensed to the sample pad and the result was read after 15 min. A negative result was indicated if only the ‘C’ band developed. Also a positive result was indicated if both ‘C’ and ‘T’ bands developed. If ‘C’ band did not develop, the result was reported as invalid and repeated with new device. The sensitivity and specificity of the test kit was 94.1 and 97.4%, respectively.

#### Quality control

The questionnaire was prepared in English language and translated to Amharic language and then back translated to English version. One week prior to data collection, the questionnaire was pre-tested on 5% of the required sample size at USAID Mulu HIV Prevention Project Southern Area Office, Ethiopia clinic (a site different from the actual study site) to ensure questions were unambiguous. Prior to the beginning of any data collection, all data collectors were trained by the principal investigator. The collected data was checked daily for consistency and accuracy. The quality of test results was maintained using the internal quality control of the test kits.

#### Data analysis

Data entry and analysis was done using SPSS version 23 computer software. Data was summarized using descriptive statistics and presented using tables and figures. Bivariate logistic regression analysis was used to assess the association between rate of infection and socio-demographic characteristics and related factors. Multivariable logistic regression analysis was performed taking together those socio-demographic and related factors with a *p*-value less than 0.25 in bivariate logistic regression analysis. Odds ratio (OR) and its corresponding 95% confidence interval (CI) was calculated to measure the strength of association. In all cases, p- value less than 0.05 was considered to be statistically significant.

#### Operational definitions

- ➢ **Consistent condom use:** the practice of using condom in every sexual intercourse.
- **➢ Female commercial sex worker:** Any female, who undertakes sexual activity with a man in return for money, irrespective of the site of operation (i.e., street, bars, home, hotel).

## Results

### Socio-demography

A total of 338 women, who attended the confidential clinic, were enrolled in this study. The mean age of the participants was 22.1 years (standard deviation (SD), 5.52; range, 15–39 years), and the majority (63.9%) were in the age category of 15–24 years. Rural residents and those with no formal education accounted for 59.2 and 30.8%, respectively (Tables 1 and 2).

**Table 1:** Distribution of *N. gonorrhoeae* by socio-demographic characteristics of FCSWs at a confidential clinic in Hawassa city, Southern Ethiopia, 2017

Socio-demographic variables	Categories	Neisseria gonorrhea				
		No. tested (%)	No. positive (%)	COR (95% CI)	AOR(95% CI)	*P*-Value
Age(in years)	15–24	216 (63.9)	9 (4.2)	2.28 (0.49–10.96)	–	–
	25–34	109 (32.2)	2 (1.8)	1		
	35 and above	13 (3.8)	0 (0.0)			
Residence before	Urban	138 (40.8)	5 (3.6)	1.22 (0.36–4.07)	–	–
	Rural	200 (59.2)	6 (3.0)	1		
Marital Status	Single	186 (55.0)	6 (3.2)	1.70 (0.20–14.45)	–	–
	Married	52 (15.4)	1 (1.9)	1		
	Widowed & Divorced	100 (29.6)	4 (4.0)	2.12 (0.23–19.52)	–	–
Educational Status	No formal education	104 (30.8)	8 (7.7)	9.16 (1.13–74.62)*	5.28 (0.60–46.57)	0.134
	Primary education	123 (36.4)	2 (1.6)	1.82 (0.16–20.33)	1.33 (0.11–15.42)	0.826
	Secondary and above	111 (32.8)	1 (0.9)	1	1	

NB: *Candidate variable for multivariable analysis at *P* < 0.25 *variable significant at *P* < 0.05

*COR* crude odds ratio, *AOR* adjusted odds ratio, *CI* confidence interval, *P-V p* -value, *1* reference

**Table 2:** Distribution of *C. trachomatis* by socio-demographic characteristics of FCSWs at a confidential clinic in Hawassa City, Southern Ethiopia, 2017

Socio-demographic variables	Categories	*Chlamydia trachomatis*				
		No. tested (%)	No. positive (%)	COR (95% CI)	AOR(95%CI)	*P*-Value
Age(in years)	15–24	216 (63.9)	17 (7.9)	1.46 (0.56–3.83)	–	–
	25–34	109 (32.2)	6 (5.5)	1		
	35 and above	13 (3.8)	0 (0.0)			
Residence before	Urban	138 (40.8)	11 (8.0)	1.35 (0.59–3.17)	–	–
	Rural	200 (59.2)	12 (6.0)	1		
Marital Status	Single	186 (55.0)	11 (5.9)	1.01 (0.28–3.83)	–	–
	Married	52 (15.4)	3 (5.8)	1		
	Widowed & Divorced	100 (29.6)	9 (9.0)	1.58 (0.42–6.25)	–	–
Educational Status	No formal education	104 (30.8)	10 (9.6)	1.86 (0.65–5.32)*	1.42 (0.31–6.59)	0.652
	Primary education	123 (36.4)	7 (5.7)	1.06 (0.34–3.24)	0.93 (0.19–4.43)	0.927
	Secondary and above	111 (32.8)	6 (5.4)	1	1	

*NB*: *Candidate variable for multivariable analysis at *P* < 0.25 *variable significant at *P* < 0.05

*COR* crude odds ratio, *AOR* adjusted odds ratio, *CI* confidence interval, *P-V p* -value, *1* reference

### Prevalence of *Neisseria gonorrhoeae* and *Chlamydia trachomatis*

The prevalence of *N. gonorrhoeae* and *C. trachomatis* was 3.3 and 6.8%, respectively. Co-infection of *N. gonorrhoeae* and *C. trachomatis* was detected in 0.6% of the FCSWs. A higher rate of *N. gonorrhoeae* (4.2%) and *C. trachomatis* (7.9%) was observed among FCSWs in the age range 15–24 years. N*. gonorrhoeae* and *C. trachomatis* infections were detected in 3.6 and 8% of urban residents, and in 7.7 and 9.6% of those FCSWs with no formal education, respectively (Tables 1 and 2).

In bivariate analysis, the prevalence of *N. gonorrhoeae* infection was shown to be higher among FCSWs with no formal education compared to those who had secondary and above educational level (COR 9.2; 95% CI 1.13–74.62). However, there was no statistically significant association between *N. gonorrhoeae* infection and site of residence, marital status, and age of the participants. In further analysis, after adjustment for those significantly associated variables using multivariable logistic regression analysis, the association between *N. gonorrhoeae* infection and educational status was not found to be statistically significant (AOR 5.3; 95% CI 0.60–46.57).

### Self-reported factors for *N. gonorrhoeae* and *C. trachomatis* infection

The exposure to different factors of *N. gonorrhoeae* and *C. trachomatis* infections among the study participants is presented in Tables 3 and 4. The majority (90.2%) of the participants responded they regularly used condom during sexual practice with their clients. Seventy nine percent of the respondents had ≥7 clients per week within the last 6 months. Most (84.9%) of the study participants were engaged in sex work for ≤5 years, and 67.8% of the respondents had first sex after the age of 15 years. About 39.1% of the study participants had a history of STIs, and 68.3% used contraceptives.

**Table 3:** Bivariate and multivariable analysis of factors associated with *N. gonorrhoeae* infection among FCSWs in Hawassa City, Southern Ethiopia, 2017

Socio-demographic variables	Categories	*Neisseria gonorrhea*				
		No. tested (%)	No. positive (%)	COR (95% CI)	AOR(95%CI)	*P*-Value
Years in sex work	< 5 yrs	287 (84.9)	8 (2.8)	1		
	> 5 yrs.	51 (15.1)	3 (5.9)	2.28 (0.56–8.51)	–	–
Place of work	Home	16 (4.8)	1 (6.3)	2.65 (0.28–25.25)	–	–
	Street	159 (47.0)	6 (3.8)	1.55 (0.43–5.63)	–	–
	Hotel / bar	163 (48.2)	4 (2.5)	1		
Age at first sex	< 15 yrs	109 (32.2)	4 (3.7)	1.21 (0.35–4.23)	–	–
	> 15 yrs	229 (67.8)	7 (3.1)	1		
Age at starting sex work	< 15 yrs	16 (4.7)	1 (6.3)	0.49 (0.14–1.74)	–	–
	15–19 yrs	214 (63.3)	5 (2.3)	1		
	> 20 yrs	108 (32.0)	5 (4.6)	1.37 (0.15–12.57)	–	–
No of customers/ week	< 7 clients	267 (79.0)	9 (3.4)	1.20 (0.25–5.70)	–	–
	> 7 clients	71 (21.0)	2 (2.8)	1		
Consistent condom use	Yes	305 (90.2)	6 (2.0)	1	1	
	No	33 (9.8)	5 (15.2)	8.89 (2.56–31.00)*	6.31 (1.61–24.86)	0.008*
Having regular sexual partner	Yes	201 (59.5)	6 (3.0)	1		
	No	137 (40.5)	5 (3.6)	1.23 (0.37–4.12)	–	–
Consistent condom use with regular partner	Yes	187 (93.0)	4 (2.1)	1	1	
	No	14 (7.0)	1 (7.1)	3.52 (0.37–33.81)*	2.56 (0.39–17.46)	0.319
Not using condom for better payment	Yes	19(5.6)	2 (10.5)	4.05 (0.81–20.23)	–	–
	No	319(94.4)	9 (2.8)	1		
Using contraceptives	Yes	231 (68.3)	6 (2.6)	1		
	No	107(31.7)	5 (4.7)	1.84 (0.55–6.16)	–	
Drinking alcohol before sex	Yes	276(81.7)	9 (3.3)	1.01 (0.21–4.80)	–	–
	No	62(18.3)	2 (3.2)	1		
Self-reported history of STIs	Yes	132 (39.1)	7 (5.3)	2.83 (0.81–9.86)*	2.83 (0.76–10.52)	0.122
	No	206 (60.9)	4 (1.9)	1	1	

NB: *Candidate variable for multivariate analysis at *P* < 0.25 *variable significant at *P* < 0.05

*COR* crude odds ratio, *AOR* adjusted odds ratio, *CI* confidence interval, *P-V p* -value, *1* reference, *STIs* sexual transmitted infections, *yrs.* years, *No* total number

**Table 4:** Bivariate and multivariable analysis of factors associated with *C. trachomatis* infection among FCSWs in Hawassa City, Southern Ethiopia, 2017

Socio-demographic variables	Categories	*Chlamydia trachomatis*				
		No. tested (%)	No. positive (%)	COR (95% CI)	AOR(95%CI)	*P*-Value
Years in sex work	< 5 yrs	287 (84.9)	19 (6.6)	1		
	> 5 yrs.	51 (15.1)	4 (7.8)	1.20 (0.39–3.69)	–	–
Place of work	Home	16 (4.8)	2 (12.5)	2.18 (0.44–10.98)	–	–
	Street	159 (47.0)	11 (6.9)	1.14 (0.47–2.76))	–	–
	Hotel / bar	163 (48.2)	10 (6.1)	1		
Age at first sex	< 15 yrs	109 (32.2)	6 (5.5)	1		
	> 15 yrs	229 (67.8)	17 (7.4)	1.37 (0.53–3.60)	–	–
Age at starting sex work	< 15 yrs	16 (4.7)	1 (6.3)	0.73 (0.29–1.72)	–	–
	15–19 yrs	214 (63.3)	13 (6.1)	1		
	> 20 yrs	108 (32.0)	9 (8.3)	0.71 (0.09–6.21)	–	–
No of customers/ week	< 7 clients	267 (79.0)	17 (6.4)	1		
	> 7 clients	71 (21.0)	6 (8.5)	1.35 (0.52–3.58)	–	–
Consistent condom use	Yes	305 (90.2)	16 (5.2)	1	1	
	No	33 (9.8)	7 (21.2)	4.86 (1.86–12.89)*	4.03 (1.06–15.31)	0.040*
Having regular sexual partner	Yes	201 (59.5)	14 (7.0)	1.07 (0.45–2.53)	–	–
	No	137 (40.5)	9 (6.6)	1		
Consistent condom use with regular partner	Yes	187 (93.0)	11 (5.9)	1	1	
	No	14 (7.0)	3 (21.4)	4.36 (1.06–17.95)*	4.01 (0.88–18.35)	0.073
Not using condom for better payment	Yes	19(5.6)	2 (10.5)	1.67 (0.36–7.71)	–	–
	No	319(94.4)	21 (6.6)	1		
Using contraceptives	Yes	231 (68.3)	15 (6.5)	1		
	No	107(31.7)	8 (7.5)	1.16 (0.48–2.84)	–	–
Drinking alcohol before sex	Yes	276(81.7)	20 (7.2)	1.54 (0.44–5.34)	–	–
	No	62(18.3)	3 (4.8)	1		
Self-reported history of STIs	Yes	132 (39.1)	12 (9.1)	1.77 (0.76–4.14)*	1.25 (0.39–3.89)	0.706
	No	206 (60.9)	11 (5.3)	1	1	

NB: *Candidate variable for multivariate analysis at *P* < 0.25 *variable significant at *P* < 0.05

*COR* crude odds ratio, *AOR* adjusted odds ratio, *CI* confidence interval, *P-V p* -value, *1* reference, *STIs* sexual transmitted infections, *yrs.* years, *No* total number

FCSWs, who did not use condom regularly, had a higher rate of *N. gonorrhoeae* infection (15.2%) compared to those who consistently used condom (2.2%) (COR 8.9; 95% CI 2.56–31.00). Similarly, the association between *C. trachomatis* infection and practice of condom use was found to be statistically significant (COR 4.86; 95% CI 1.86–12.89). Further, FCSWs, who did not use condom with regular partners, were at higher odds of having *C. trachomatis* infection compared to those who consistently use condom (COR 4.4; 95% CI 1.06–17.95).

In multivariable logistic regression analysis, FCSWs, who did not use condom regularly in the last 6 months, were at higher risk of acquiring *N. gonorrhoeae* (AOR 6.3; 95% CI 1.61–24.86,**)** and *C. trachomatis* (COR 4.28; 95% CI 1.06–15.31) infections compared to those who regularly used condom. However, there was no statistically significant association between *N. gonorrhoeae /C. trachomatis* infection and other variables such as place of work, using contraceptives, drinking alcohol before sex, and age at starting sex work.

## Discussion

*C. trachomatis* and *N. gonorrhoeae* infections are an important public health problem worldwide. Most developed countries have implemented specific chlamydial and gonococal infection control programs that include case management, opportunistic screening of high risk groups, and annual screening of sexually active women aged < 25 years. However, in developing countries like Ethiopia where laboratory facilities are limited, the management of STIs is still based on non-specific syndromic approach, which results in over-prescription of drugs. Further, the epidemiology of STIs is not well described in Ethiopian settings so as to design and implement evidence-informed intervention strategies. This study aimed to assess the prevalence and associated risk factors of *C. trachomatis* and *N. gonorrhoeae* infections among FCSWs.

In this study, the prevalence of *N. gonorrhoeae* and *C. trachomatis* among FCSWs was 3.3 and 6.8%, respectively. The observed rate of *C. trachomatis* infection was in agreement with results reported in Congo (8.4%) [13], Kenya (8%) [14], Netherlands (7.1%) [15], and Barcelona (4.7%) [16]. However, a contrasting lower result was reported in Kenya (3.1%) [13], and higher results were reported in Senegal (20%) [17] and Côte d’Ivoire (11.8%) [18]. These difference in prevalence may be due to a varying distribution of risk factors in different countries and dissimilar laboratory methods employed.

Moreover, the observed rate of *N. gonorrhoeae* in the current study was consistent with findings reported in Mexico (3.7%) [19], Netherlands (2.6%) [15], and Peru (1.6%) [20]. However, as the studies used highly sensitive laboratory techniques such as polymerase chain reaction (PCR) and nucleic acid amplified tests (NAAT) [15, 19, 20], the epidemiological significance of gonorrhea might be higher in our setting. In contrast to our finding, higher rates of gonorrhea were reported in Ghana (33.7%) [21], Senegal (22%) [17], and Côte d’Ivoire (9.1%) [19], and a lower rate was reported in Kenya (1.1%) [13]. The disparities in prevalence may be related to difference in composition of the studied population, distribution of risk factors, and effectiveness of STIs intervention programs [22].

The observed rate of co-infection of *N. gonorrhoeae* and *C. trachomatis* in the current study (0.6%) was in agreement with the result reported in India (1.1%) [23]. The occurrence of co-infection might be due to the fact that these bacteria share mode of transmission, and the presence of one infection could facilitate the acquisition of another [24].

It was also observed that the practice of using condom influenced the infection status with *N. gonorrhoeae* and *C. trachomatis*, where FCSWs, who regularly used condom, were more protected against the infections. This finding was also supported by the study from China [25].

Both *N. gonorrhoeae* and *C. trachomatis* infections were detected more frequently among FCSWs in the age range 15–24 years, though difference by age was not statistically significant. This finding was also compatible with the result reported in a similar study from China [26]. In contrast to our finding, infections were more likely detected among participants in the age range ≥ 35 years in Korea [27]. A higher prevalence of *N. gonorrhoeae* and *C. trachomatis* among younger age group in our study might be related to having more number of sex clients per week and a lower level of awareness to STIs prevention [28].

As reported elsewhere [26], FCSWs, who got divorced or widowed, had a higher rate of infection with *N. gonorrhoeae* and *C. trachomatis* compared to those who were never married. Lack of social and economic support might expose them to engage in sex work and experience sexual violence, which increases the risk of contracting STIs [29]. Further, in agreement to a study in Argentina [30], FCSWs, who inconsistently used condom with their regular partners, had higher rate of *N. gonorrhoeae* and *C. trachomatis* infections compared to those who consistently used condom.

Our study has some limitations. First, we used a rapid diagnostic test for detection of *C. trachomatis* and culture for *N. gonorrhoeae,* which are less sensitive than PCR assays to accurately report the burden of these infections. Second, some of the assessed variables are confounded by recall bias or social desirability bias, leading to inaccurate response. Third, as the study investigated FCSWs, who attended the confidential clinic, the generalizability of the finding is also limited. Fourth, antibiotic resistance testing was not performed in the current study due to lack of resources. Fifth, our study considered small sample size, which limits the precision of our prevalence estimate. Despite these shortcomings, this study generated relevant information in Ethiopian setting where epidemiological data on *N. gonorrhoeae* and *C. trachomatis* infection is scarce.

In conclusion, this epidemiological study documented a high prevalence of *N. gonorrhoeae* and *C. trachomatis* infections among FCSWs. The study also showed that FCSWs, who inconsistently used condom, were at higher risk of contracting *N. gonorrhoeae* and *C. trachomatis* infections. Therefore, the findings of this study call for intervention measures that reduce the transmission of the infections. We suggest to the Ministry of Health the importance of providing screening service for FCSWs to detect and mange asymptomatic cases. Further, the Regional Health Bureau, health facilities in Hawassa City, and the confidential clinic need to emphasize improving the current condom promotion activities.
